# Predicting tumor cell line response to drug pairs with deep learning

**DOI:** 10.1186/s12859-018-2509-3

**Published:** 2018-12-21

**Authors:** Fangfang Xia, Maulik Shukla, Thomas Brettin, Cristina Garcia-Cardona, Judith Cohn, Jonathan E. Allen, Sergei Maslov, Susan L. Holbeck, James H. Doroshow, Yvonne A. Evrard, Eric A. Stahlberg, Rick L. Stevens

**Affiliations:** 1 Computing, Environment and Life Sciences, Argonne National Laboratory, Lemont, IL, USA; 2 Computation Institute, The University of Chicago, Chicago, IL, USA; 3 Center for Nonlinear Studies, Los Alamos National Laboratory, Los Alamos, NM, USA; 4 Computer Science, Los Alamos National Laboratory, Los Alamos, NM, USA; 5 Computation Directorate, Lawrence Livermore National Laboratory, Livermore, CA, USA; 6 Department of Bioengineering and Carl R. Woese Institute for Genomic Biology, University of Illinois at Urbana-Champaign, Urbana, IL, USA; 7 Developmental Therapeutics Branch, National Cancer Institute, Frederick, MD, USA; 8 Data Science and Information Technology Program, Frederick National Laboratory for Cancer Research, Frederick, MD, USA

**Keywords:** Machine learning, Deep learning, Combination therapy, in silico drug screening

## Abstract

**Background:**

The National Cancer Institute drug pair screening effort against 60 well-characterized human tumor cell lines (NCI-60) presents an unprecedented resource for modeling combinational drug activity.

**Results:**

We present a computational model for predicting cell line response to a subset of drug pairs in the NCI-ALMANAC database. Based on residual neural networks for encoding features as well as predicting tumor growth, our model explains 94% of the response variance. While our best result is achieved with a combination of molecular feature types (gene expression, microRNA and proteome), we show that most of the predictive power comes from drug descriptors. To further demonstrate value in detecting anticancer therapy, we rank the drug pairs for each cell line based on model predicted combination effect and recover 80% of the top pairs with enhanced activity.

**Conclusions:**

We present promising results in applying deep learning to predicting combinational drug response. Our feature analysis indicates screening data involving more cell lines are needed for the models to make better use of molecular features.

## Background

Deep learning has already revolutionized the fields of computer vision, robotics, gaming, and natural language processing. It is rapidly making strides in genomics, medical diagnosis, and computational chemistry. At the heart of deep learning is a set of generalizable techniques that thrive with large-scale data in inferring complex relationships. While emerging neural networks are outperforming state-of-the-art models in predicting a wide range of molecular properties, to date, their use has been limited to single molecules due to the lack of training data.

The recently published NCI-ALMANAC resource [1] will change that. It offers a promising look at the combined effect of small molecules, through systematic evaluation of 104 FDA-approved oncology drugs. All drug pairs were screened at multiple concentrations against the NCI-60 panel of human tumor cell lines, resulting in 3 million data points. Twenty one percent of the drug combinations were revealed to have greater than additive activity in inhibiting cell line growth. Such synergistic interaction of small molecules may be rooted in the hypothesis that they combine to overcome the inherent heterogeneity of tumors and prevent emergence of drug resistance in cell subpopulations.

In this paper, we model the combined activity of anticancer drugs with deep neural networks. One advantage of this approach over traditional machine learning is the joint training of intermediate features and final prediction. We first collect multiple types of molecular and drug features and pass them through separate featurization submodels. The encoded features from these models are then concatenated to predict growth inhibition. Our best model achieves a mean absolute error below 10%, with coefficient of determination *R*^2^ of 0.94 and Pearson correlation coefficient of 0.97 in 5-fold cross validation. While paired drug response prediction at this scale has not been attempted before to our knowledge, most machine learning models, in comparison, achieve less than 0.9 *R*^2^ for a wide range of single molecule regression benchmarks [2], with the exception of quantum mechanics tasks [3].

We further analyze feature importance and model bias. We show that, with only 60 cell lines, all three molecular feature types examined provide marginal benefit, whereas most of the predictive capacity resides in drug descriptors generated by the Dragon software [4]. We visualize growth prediction errors with aggregated plots from the perspective of cell lines or drug pairs and find no obvious systematic bias.

Finally, we test the utility of our model in virtual screening of drug pairs. With a customized combination score derived from predicted growth fractions, we are able to identify the majority of synergistic drug pairs.

### Related Work

Early machine learning applications in cancer and drug discovery focused on only one type of genomic profiles [5–8]. In 2012, NCI and the DREAM consortium launched a drug sensitivity challenge that integrated multiple omics measurements. Among the 44 community-based approaches, top performing models included Bayesian multitask multiple kernel learning [9] and ensemble based methods [10]. In another open NCI-DREAM challenge, 31 methods competed on predicting drug pair activity. Although these methods performed significantly better than chance, none was near-optimal [11]. Since both challenges focused on ranking order as the evaluation metric, their results were not directly comparable to this study. Also, properties of the drugs were not used in either challenge as input; they probably would not have helped, due to the small sample sizes (29 single drugs and 91 drug pairs, respectively).

Menden et al. [12] used both cell line and drug features to predict the half maximal inhibitory concentration (IC_50_) on the screening results from the Genomics of Drug Sensitivity in Cancer (GDSC) project [13]. They achieved *R*^2^ values of 0.72 and 0.64 on cross validation and holdout test data, respectively. It was still early days for the rebirth of neural networks. The machine learning models in that study combined random forests and shallow networks with no more than 30 hidden units.

Since then, the transforming impact of deep learning has spread to the fields of computational biology [14] and computational chemistry [15]. A defining moment was a deep neural network winning the Kaggle challenge on molecular activity [2]. More molecular-based datasets and prediction tasks were recently curated in the MoleculeNet benchmark and the DeepChem open source library. Multiple machine learning methods were tried to predict targets in four different activity categories. In the three categories with regression tasks, the best validation *R*^2^ ranged 0.61–0.87 for physical chemistry, 0.45–0.51 for biophysics, and around 0.99 for quantum mechanics [3].

## Methods

In this section, we will first describe the NCI-ALMANAC drug pair response data which is the prediction target of our computational model. The model takes into account properties of both tumor cell lines and drugs; we will also cover these two sets of input features.

### Drug pair screen data

Cancer is an extremely complex disease. A single tumor can develop on the order of 100 million coding region mutations that potentially foster drug resistance [16]. To combat this enormous diversity, combination therapies are developed to interact with multiple targets simultaneously. While some promising drug combinations can be identified based on known cell biology, much of the multidrug interaction mechanisms remain unknown. In an effort to systematically examine the combination efficacy of 104 FDA-approved anticancer drugs, the NCI-ALMANAC resource catalogs in vitro screen results of their pairwise combinations against the NCI-60 cell lines [1].

In this study, we worked with a subset of the NCI-ALMANAC data. We included 54 of the 104 drugs for which we were able to compute molecular descriptors with the Dragon software. We left out one of the 60 cell lines because of significant missing information in molecular characterization. We also filtered the screen results based on the NCI-defined quality control standards. These criteria narrowed down the number of experiments from 304,549 in the original dataset to 85,303.

**Fig. 1:**
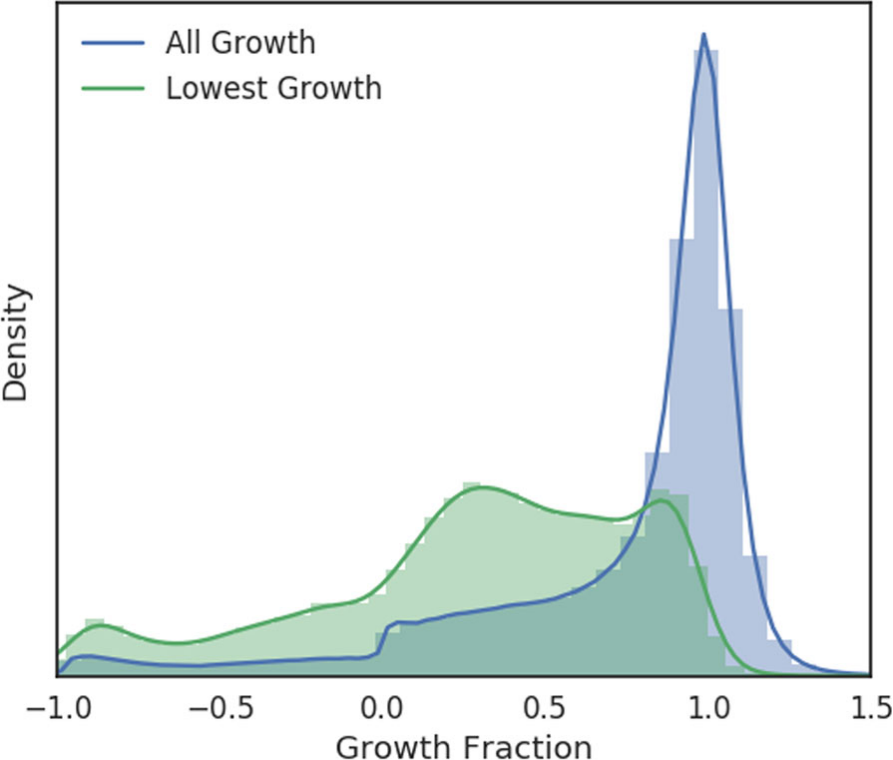
Normalized histograms of cell line growth fractions under drug pair treatments. The blue histogram represents all growth fractions. For every (cell line, drug 1, drug 2) tuple, there are multiple cell line growth values corresponding to the different dose level combinations of the two drugs; the distribution of the lowest growth fraction for each tuple is depicted in green

In the NCI-ALMANAC project, each drug experiment was tested with multiple concentrations: single drugs were tested at 3 or 5 concentrations, and drug pairs were tested at 3 ×3 or 5 ×3 combination matrices. Most of the concentrations were chosen to be below the levels corresponding to FDA-approved clinical doses. The response value was relative tumor growth described in the standard NCI-60 testing protocol [17]. In this study, the growth inhibition percentage was converted to fraction, ranged from −1 to slighly above 1, with −1 representing 100% lethality, 0 representing total inhibition, and 1 representing unabated growth compared to control tumor tissue. The raw growth inhibition data contain cases where the drugs appear to have enhanced tumor growth (see distribution in Fig. 1) with greater than 1 growth fraction; most of these were not considered to be real and capped at 1.

We did not include drug concentration as an input feature in our computational model. Instead, we tried to predict the best growth inhibition seen in any experiment for a given drug or drug pair. The main motivation was to reduce training data imbalance and focus on the more effective dose combinations, knowing most of the doses tested were within FDA-approved ranges. As shown in Fig. 1, the original distribution of screen results was heavily tilted to the nonresponse end; after removing the less effective dose combinations, the distribution became less imbalanced.

The NCI-ALMANAC project defined a ComboScore to quantify the benefit of combining two drugs. For a cell line and drug combination, this score was the sum of the differences in observed versus expected growth fractions over all dose combinations. In this paper, we use a modified version of ComboScore that only considers the concentrations that led to the best growth inhibition for a given cell line and drug combination. Following the notation in the NCI-ALMANAC paper, we use $y_{i}^{AB}$ to denote the lowest growth fraction for the *i*^*th*^ cell line exposed to drug pair *A* and *B*, considering all dose combinations experimented for that pair. We call $y_{i}^{AB}$ “MinComboGrowth”. Similarly, let $y_{i}^{A}$, $y_{i}^{B}$ be the lowest growth fractions when only exposed to drug *A* or drug *B*, respectively. The modified expected growth fraction for the combination is:

$$ z_{i}^{AB} =\left\{ \begin{array}{ll} \min\left(y_{i}^{A}, y_{i}^{B}\right),& \text{if}\;\; y_{i}^{A} \leq 0 \text{ or}\ y_{i}^{B} \leq 0\\ \tilde{y}_{i}^{A} \cdot \tilde{y}_{i}^{B}, & \text{otherwise} \end{array}\right. $$

where $\tilde {y}_{i}=\min (y_{i}, 1)$ truncates the growth fraction at 1. The modified combination score, termed “BestComboScore”, is thus:

$$C_{i}^{AB} = \left(y_{i}^{AB} - z_{i}^{AB}\right) \times 100 $$

### Molecular characterization

The NCI-60 human tumor cell line panel was developed in the 1980s and has been widely used as a tool for anticancer drug screen [18]. Each of the cell lines has been extensively profiled, using a variety of high-throughput assays, for gene expression, exome sequence, mutations, DNA methylation, microRNA expression, protein abundance, protein modification, enzyme activity, and metabolomics. In these molecular datasets, gene expression has been demonstrated to be among the best predictors for cancer drug response [9]. Protein abundance and microRNA expression profiles are two emerging assay types that are increasingly recognized as informative features for their role in anticancer regulation [19–24]. We therefore included these three datasets as input features.

**Gene expression** The gene transcript expression levels were downloaded from NCI’s CellMiner [25] using the version averaged from five microarray platforms. Each cell line is characeterized by 25,723 gene features.

**microRNA expression** microRNA expression levels were also downloaded from CellMinor. This dataset contains 454 feature columns for each cell line.

**Protein abundance** Proteomics data was downloaded from the NCI-60 Proteome Database [26, 27]. This dataset reports the protein abundance levels for a subset of proteins in 59 cell lines. The data for the problematic MDA-N cell line is not available. This dataset combines 8097 proteins and 1663 kinases into 9760 features for each cell line.

### Drug descriptors and fingerprints

Dragon is a commercial software package for computing molecular descriptors that can be used for quantitative structure–activity relationship (QSAR) modeling or virtual screening of chemical databases. The software generates 30 categories of molecular descriptors (e.g., ring descriptors, topological indices, path counts, atom pairs, drug-likeness) and two different types of fingerprints (path fingerprint and extended connectivity fingerprint).

We downloaded 2D structure data for the 104 FDA-approved drugs from the NCI ALMANAC database. We were able to use Dragon (version 7.0) to generate descriptors for 54 of these drugs. Among the 5270 descriptors and fingerprints generated for each drug, many columns had missing values. They included 3D descriptors (expected) and other categories such as functional group counts, edge adjacency indices, atom pairs 2D, and CATS 2D. We removed descriptor columns for which at least 90% of the drug rows were missing. This reduced the descriptor matrix dimension to 54×3809.

### Data preprocessing

The gene expression and microRNA data downloaded from CellMiner were already log(*x*+1) transformed. We applied the same transformation to protein abundance data. The drug descriptors had varied ranges (e.g., binary for finger prints, hundreds for molecular weight), and we did not transform them. We built the data generators feeding our neural network model with multiple options for data imputation and scaling. For the experiments presented in this paper, we first filled the missing values with the mean over cell lines and then used min-max scaling to normalize each feature to the [0,1] range.

### Neural network architecture

**Fig. 2:**
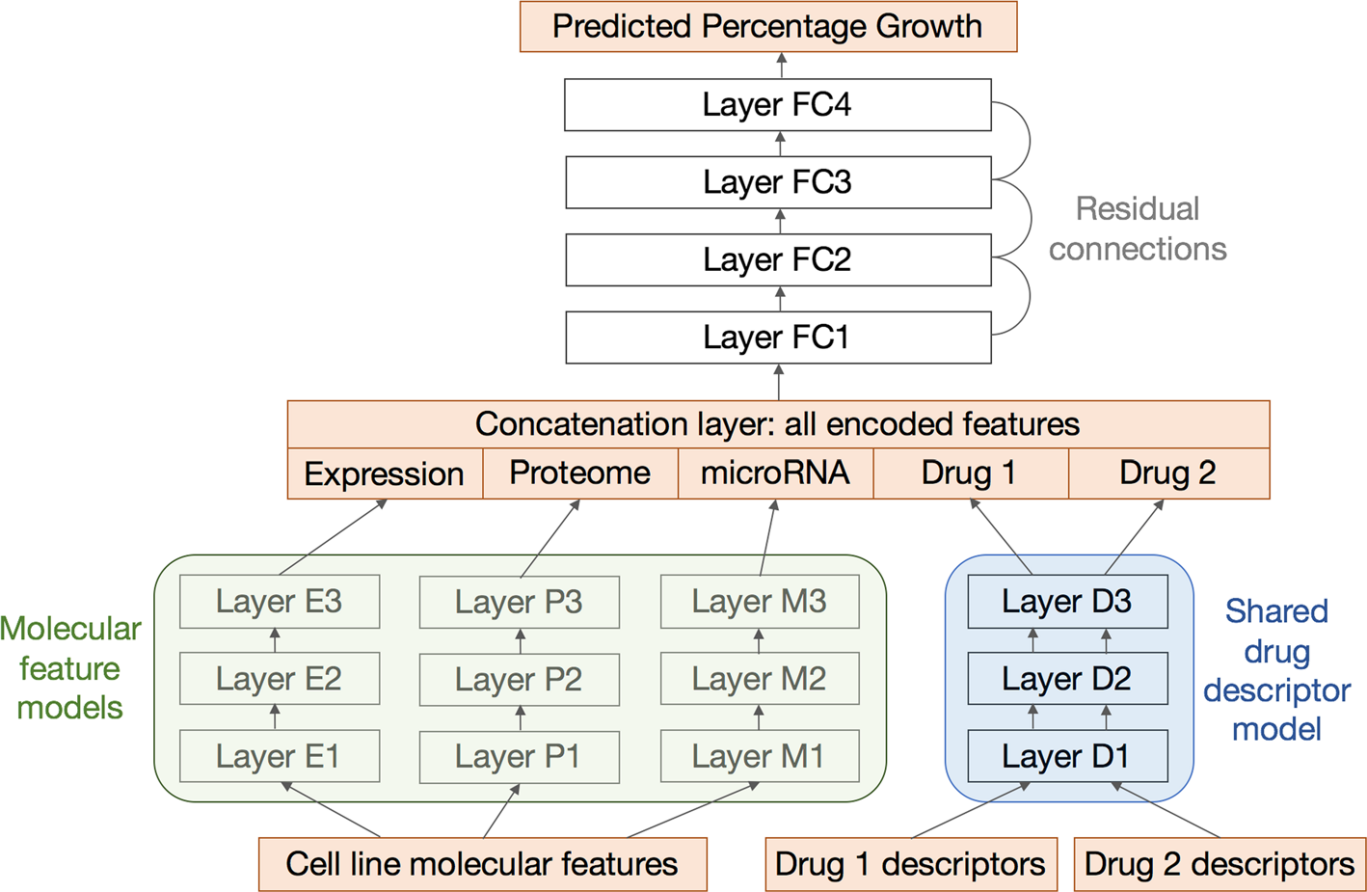
Neural network architecture. The orange square boxes, from bottom to top, represent input features, encoded features, and output growth values. Feature models are denoted by round shaded boxes: green for molecular features and blue for drug features. There are multiple types of molecular features that are fed into submodels for gene expression, proteome, and microRNA. The descriptors for the two drugs share the same descriptor model. All encoded features are then concatenated to form input for the top fully connected layers. Most connecting layers are linked by optional residual skip connections if their dimensions match

Our neural network model takes the preprocessed features for a cell line and drug combination as input and generates a scalar prediction on growth inhibition. The architecture of a typical network instance is depicted in Fig. 2. This architecture consists of two levels to simultaneously optimize for feature encoding and response prediction.

**Feature encoding submodels** Each molecular assay dataset is fed into a separate feedforward network to encode a new vector representation of that feature type. The drug descriptor submodel is also a feedforward network. Because the two drug slots are symmetric, the mechanism that encodes the first drug should be reused for the second. Therefore, all the weights and layers are shared between the two drugs. The drug pair screen data cover single drug experiments as well. These data points are easily accommodated by replicating the same set of descriptors to the two drug slots.

**Growth prediction submodel** All the encoded molecular and drug features are optionally concatenated to form the input for growth prediction. This submodel is a feedforward network with typically more layers than the feature encoding networks.

All the networks are fully connected within themselves. The number of layers and number of neurons in each layer are hyperparameters controlled manually or by an external script. To improve training efficiency, the adjacent layers in each network are optionally connected by a residual operator [28] when their dimensions match. The model was implemented with Keras [29] and trained to optimized mean squared error (MSE).

## Results

We measured the performance of the drug pair response model with 5-fold cross validation. Each pair measurement was treated as an independent data point to create folds. The folds were stratified; that is, we first binned the growth fractions evenly into 5 classes and made folds by preserving the percentage of samples for each class.

### Hyperparameter search

We have not done an comprehensive hyperparameter sweep. The current best parameter set and the insights we gained from about thousands of runs are listed as follows:

- The feature encoding networks should have two or more hidden layers.
- The growth prediction network should have three or more hidden layers.
- Each hidden layer should have more than 1000 neurons.
- Residual connections reduce training time and occasionally improve final validation loss; thus a network with the same number of neurons in neighboring hidden layers is preferred to a pyramid-shaped network.
- Each hidden layer should have more than 1000 neurons.
- The model should be trained for at least 100 epochs.
- The Adam optimizer is recommended.
- Reducing learning rate on plateau improves validation loss.
- Larger learning rate improves training speed without much loss in accuracy when used in combination with larger batches and a warm-up schedule [30].

### Feature importance

We tested our model on different combinations of feature categories to assess their relative importance. The average metrics from 5-fold cross validation runs are listed in Table 1.

**Table 1:** Cross validation results from feature combination experiments

Molecular features	Drug features	MSE	MAE	*R* ^2^
Baseline	Baseline	0.5253	0.5709	-1.001
One-hot encoding	One-hot encoding	0.2448	0.3997	0.1269
Gene expression	One-hot encoding	0.2447	0.3999	0.1272
Gene expression	500-dimensional noise	0.2450	0.4008	0.1271
One-hot encoding	Dragon7 descriptors	0.0292	0.1086	0.8892
Proteome	Dragon7 descriptors	0.0303	0.1117	0.8844
microRNA	Dragon7 descriptors	0.0275	0.1050	0.8952
Gene expression	Dragon7 descriptors	0.0180	0.0906	0.9364
Gene expression, microRNA, proteome	Dragon7 descriptors	**0.0158**	**0.0833**	**0.9440**

The boldface row represents the best cross validation

The baseline performance was established by comparing random pairs of growth fractions. This is equivalent to guessing by random selection from the real distribution. The mean absolute error (MAE) between any two growth fractions is 0.53, and *R*^2^ for this baseline setting is close to −1 (as defined in the Scikit-learn Python package [31], *R*^2^ is allowed to be negative).

We then encoded the molecular and drug features with simple one-hot or fixed random vectors. This was intended to assess whether the molecular assays or drug descriptors provided additional predictive power over mere identifiers. With one-hot encoded drugs, *R*^2^ stayed around 0.12, irrespective of how the cell lines were encoded. When the Dragon descriptors were used, *R*^2^ jumped above 0.88. However, among these models, the prediction improvement was at best marginal with extra molecular data. Finally, the best model performance (*R*^2^=0.94) was achieved with all three types of molecular assays along with the Dragon descriptors. The Pearson’s correlation for this model is 0.972 and the Spearman’s rank correlation is 0.965.

### Error analysis

While the overall *R*^2^ is high for molecular prediction tasks, we sought to understand if there were systematic biases in prediction error. One way to do that is to plot the aggregated errors from the perspective of cell lines or drug pairs. In other words, consider a 3D grid (cell line × drug 1 × drug 2) of drug response experiments, we can flatten the corresponding 85,303 validation errors along the cell line axis or onto the drug pair plane.

**Fig. 3:**
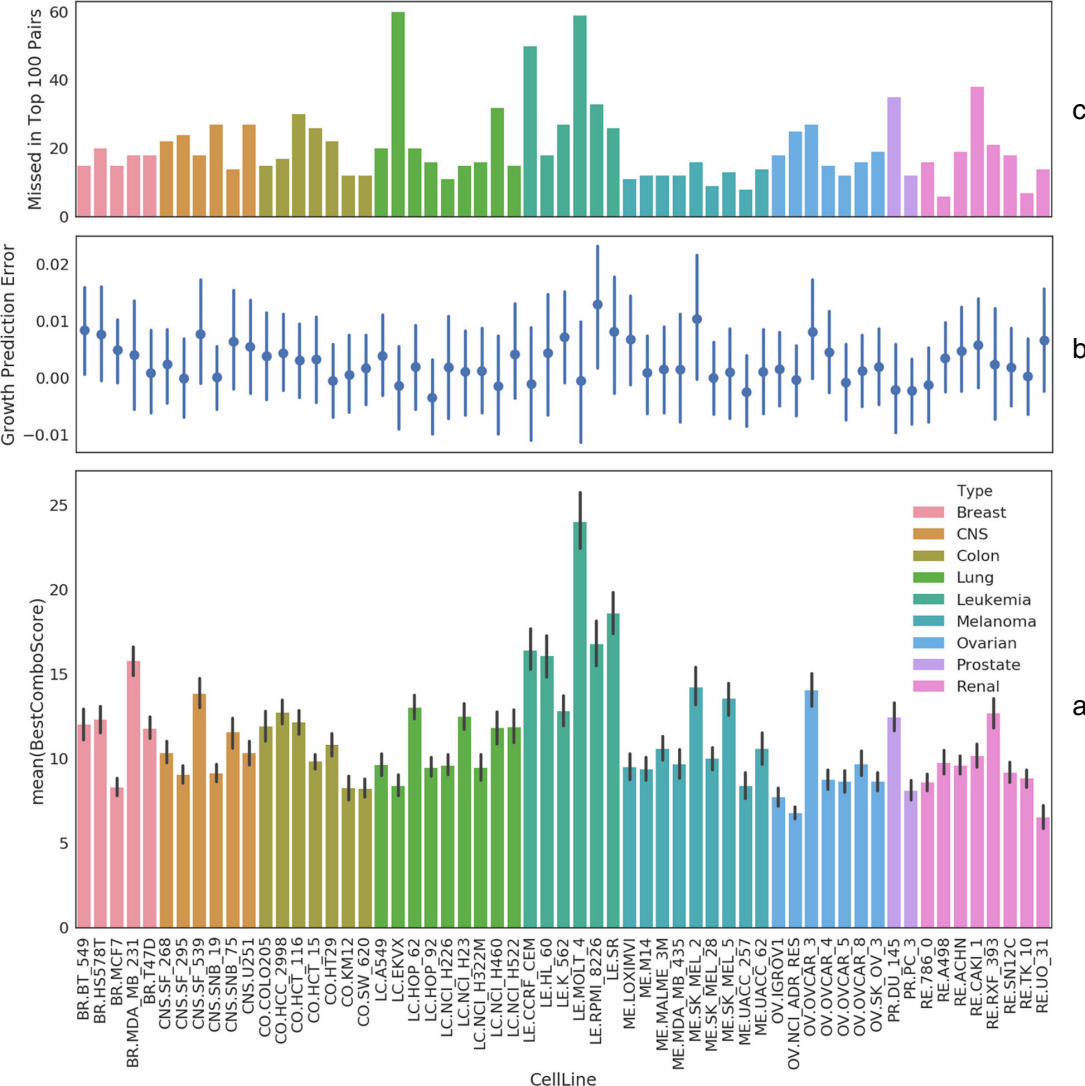
Cell line views of drug combination effect, growth prediction error, and ranking error. **a**, the bar plot depicts the drug combination scores averaged over all drug pairs for each cell line. 95% confidence intervals are indicated by black vertical lines. **b**, errors in predicted growth fractions, aggregated over all drug pairs for each cell line, are shown with standard deviation. **c**, first, the top 100 drug pairs are determined for each cell line based on the best ComboScore among all experimented combinations of dose levels; a second top 100 list is derived from predicted growth fractions for single and paired drug responses; the difference between these two sets in terms of unique members in each set is shown for each cell line in the bar plot

**Cell line views** We first visualize mean BestComboScore in a bar plot in Fig. 3a. This gives us some idea about which cell lines tend to have drug pairs with enhanced activity. For example, all but one leukemia cell lines (the middle green block) are near the top in terms of this metric. We then stack on top of this plot two visualizations of prediction errors: (1) Fig. 3b shows there are no cell lines for which the model did particularly good or bad. Growth fraction prediction errors mostly cancel out near 0. There is also no apparent correlation between this error and BestComboScore either. (2) Fig. 3c shows how the model fares in ranking the drug pairs for each cell line in terms of BestComboScore. The model on average misses 20 of the top 100 drug pairs. 75% of the cell lines have the predicted top 100 list at least 75% correct. Rank prediction is more than half wrong for only two cell lines (LC.EKVX and LE.MOLT_4).

**Fig. 4:**
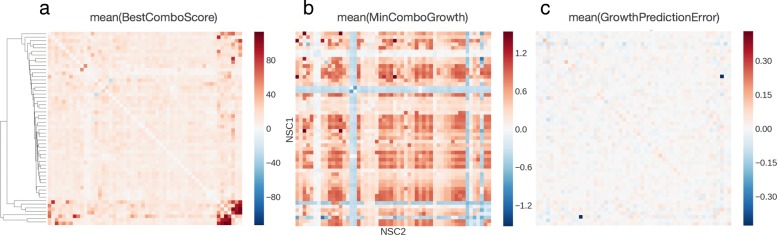
Heatmap views of combination effect, growth fraction, and growth prediction error for drug pairs. **a**, each heatmap cell represents the average, across cell lines, of the best ComboScore among different dose combinations for each drug pair. The rows and columns are drugs ordered by hierarchical clustering based on vector correlation. **b**, each cell represents the mean growth fraction for a drug pair across cell lines. The diagonal elements were filled with growth fractions from single drug experiments. **c**, each cell represents the mean difference between predicted and experimental growth fracitons for each drug pair

**Drug pair views** We first attempt to find drugs that combine well across all cell lines. Fig. 4a sorts the drugs in a heatmap based on hierarchical clustering of BestComboScore. The heatmap displays no striking pattern in average BestComboScore except the lower right corner. This corner consists of 7 drugs separated into two small groups. Any drug from group A (mithramycin, paclitaxel, dactinomycin, docetaxel) combines well with a member from group B (valrubicin, tretinoin, tamoxifen) in terms of overall enhanced activity across the cell lines. Fig. 4b then checks if the mutually enhancing drug pairs also tend to be the ones that strongly inhibit tumor growth. This correlation turns out to be weak except for the aforementioned 7 drugs. Finally, Fig. 4c examines drug pair bias in growth prediction. Only one pair (mitotane, valrubicin) stands out with −0.43 mean growth fraction error. Further examination of the training data reveals that only 4 cell lines have screen data that passed NCI’s quality control for this particular drug pair.

### Virtual screening

An important use of drug response models is in high-throughput virtual screening. If a machine learning model is accurate, it can be run in inference mode as a computational funnel to reduce screening cost. In a preliminary analysis of this flavor, we looked at how our model might be used to identify promising drug pairs in Table 2.

**Table 2:** Drug pairs with top combination scores across many cell lines

Rank	Drug pair	Predicted drug pair
1	(idarubicin, amifostine)	(idarubicin, amifostine)
2	(epirubicin, amifostine)	(epirubicin, amifostine)
3	(idarubicin, epirubicin)	(idarubicin, epirubicin)
4	(idarubicin, covidarabine)	(idarubicin, covidarabine)
5	(epirubicin, idarubicin)	(epirubicin, idarubicin)
6	(idarubicin, imiquimod)	(idarubicin, imiquimod)
7	(epirubicin, imiquimod)	(epirubicin, imiquimod)
8	(epirubicin, dexrazoxane)	(epirubicin, covidarabine)
9	(epirubicin, covidarabine)	*(epirubicin, cyclophosphamide)*
10	(idarubicin, allopurinol)	(idarubicin, allopurinol)

We first computed a list of top 100 drug pairs for each cell line using the BestComboScore derived from real growth data. We then pooled these lists from all cell lines and ranked the drug pairs by frequency. The top 10 promising drug pairs were compared to a predicted version derived from model inferred growth fractions (by merging the 5-fold cross validation results) following the same process. The resulting lists turned out to be 90% identical, with the predicted version missing (epirubicin, dexrazoxane) and overpredicting (epirubicin, cyclophosphamide).

## Discussion

Recently, much effort has been placed on the development of machine learning models that could provide a cost-effective proxy for measuring tumor drug response. While most previous studies focus on single drug screening data with classical machine learning methods, the release of the NCI-ALMANAC database has made it possible to systematically model drug pair activity with deep neural networks more suited for large-scale data. We presented a network architecture that jointly learns feature encoding and growth inhibition.

Although the model presented here gives promising results in predicting tumor cell line growth and ranking synergistic drug pairs, there is much room for improvement. For instance, the contribution of molecular features could potentially increase with inclusion of more cell lines. Another source of information we have not fully used is drug concentration. We are looking to integrate multiple single drug screening studies into a unified dose response prediction framework. Auxiliary properties such as tumor type and drug mechanism of action can also be added as auxiliary prediction targets in a multitasking model. To understand the generalizability of drug response models, more systematic cross validation schemes are needed to analyze their predictive capacity for new drugs, cell lines, and across studies.

## Conclusions

We have presented a simple, two-stage deep neural network model for predicting drug pair response. It supports multiple types of tumor features and can be easily extended to accommodate more than two drugs in combination therapy. The model achieves good performance (*R*^2^=0.94) with no strong bias toward cell lines or drug pairs. The initial result also looks promising on picking out drug pairs with consistently greater than single-agent efficacy across many cell lines. Our experiments on input features suggest that the model does not merely remember drug combinations. Rather, the drug descriptors improve *R*^2^ by 0.81. On the other hand, the predictive value of molecular assays was difficult to establish, although this will likely change if more tumor samples are included in training.

Several directions for future research seem particularly appealing. First, we plan to incorporate more drug features such as concentration, SMILES strings, molecular graph convolution [32] and atomic convolution [33] into the model. Second, we are considering other tools for calculating descriptors and fingerprints to cover the entire set of drugs; that way we will have enough data points to create a holdout set for testing. Third, we are looking at semisupervised learning methods for encoding molecular features with external gene expression and other types of data. Fourth, we will continue to explore advanced network architectures and perform rigorous hyperparameter optimization with the scalable deep learning framework we are developing.
